# Single-cell virtual knockout network perturbation prioritizes candidate regulators of beta-cell functional failure in type 2 diabetes

**DOI:** 10.3389/fendo.2026.1924512

**Published:** 2026-09-21

**Authors:** Shiyu Yang, Yunhao Li, Yunyi Yang, Xinyi Fu, Jiayi Jiang, Chunyan Wang, Fang Fu, Min Zheng, Xiaoli He, Yanming He, Zheng Yao

**Affiliations:** 1 Department of Endocrinology, Yueyang Hospital of Integrated Traditional Chinese and Western Medicine, Shanghai University of Traditional Chinese Medicine, Shanghai, China; 2 Department of Endocrinology, Rheumatology and Nephrology, Haikou Hospital of Traditional Chinese Medicine, Haikou, China

**Keywords:** beta cell, beta-cell dysfunction, gene regulatory network, pancreatic islet, scTenifoldKnk, single-cell RNA sequencing, type 2 diabetes, virtual knockout

## Abstract

**Background:**

Progressive pancreatic beta-cell dysfunction is a central feature of type 2 diabetes (T2D), but disease-associated transcriptional changes alone cannot distinguish likely regulatory drivers from secondary stress markers. Single-cell transcriptomics resolves beta-cell heterogeneity across disease states, while virtual-knockout network analysis estimates transcriptional effects after perturbing selected genes. We combined human islet single-cell RNA sequencing, beta-cell functional-state modeling, candidate scoring, scTenifoldKnk virtual knockout, and external validation to prioritize regulators associated with T2D-related beta-cell functional failure.

**Methods:**

We analyzed the official processed GSE221156 beta-cell matrix at two levels. Cell-level analyses described beta-cell states, whereas donor-level pseudobulk models covered 48 donors and adjusted for sex, age, BMI, sequencing chemistry, and ethnicity. We then connected a prior-free genome-wide screen to an independent scTenifoldKnk network-evidence analysis. The fixed 14-gene panel was tested against random panels matched for expression, detection, and network in- and out-strength. For the scale experiment, each network contained 200, 500, or 1,000 cells, and each setting used 3, 5, or 10 independently inferred networks across three seeds. Expression evidence came from GSE153855, GSE81608, GSE101207, and GSE50244; orthogonal perturbation evidence came from GSE52258, GSE230728, primary-human-pseudoislet PCSK1 CRISPR data, and quantitative PCSK2 and SLC2A2 studies. A 2,000-cell point was additionally evaluated at the primary 10-network setting.

**Results:**

GSE221156 captured substantial beta-cell heterogeneity and a redistribution of cell states. Of 26,164 expressed genes, 17,989 passed pseudobulk filtering, and 674 (3.75%) differed significantly between T2D and ND after adjustment. Larger GRNs ranked ATF4, DKK3, SLC30A8, PCSK1, and SLC2A2 highest within the prespecified 14-gene panel. The fixed 14-gene panel also exceeded 200,000 expression-, detection-, and network-strength-matched random panels (observed mean 0.619; null mean 0.578; empirical P = 0.0111). Evidence varied across individual candidates, and none survived correction across the 14 genes. Scale mattered: median cross-seed whole-profile Spearman correlation rose from 0.265 with 200 cells and 3 networks to 0.884 with 1,000 cells and 10 networks and reached 0.913 with 2,000 cells and 10 networks. Across three external beta-cell cohorts, DKK3, PCSK2, SLC2A2, and ASCL2 retained concordant directions.

**Conclusions:**

By anchoring network perturbation to donor-level disease signals, our framework maps regulatory vulnerability in T2D beta cells. The matched-null analysis identifies collective network information in the fixed 14-gene panel beyond expression and connectivity. Within this collectively enriched panel, the expanded GRNs place ATF4, DKK3, SLC30A8, PCSK1, and SLC2A2 at the leading ranks, defining focused candidates for experimental testing.

## 1 Introduction

Type 2 diabetes develops when pancreatic beta cells can no longer maintain enough insulin secretory output under chronic metabolic demand. Insulin resistance often appears before overt diabetes, but sustained hyperglycemia depends strongly on progressive beta-cell failure (1, 2). This failure involves several linked processes: loss of mature beta-cell identity, altered glucose sensing, impaired insulin processing and secretion, mitochondrial and metabolic stress, ER stress, oxidative stress, and dedifferentiation-like or dysfunctional beta-cell states (3–10).

Single-cell RNA sequencing allows these processes to be examined at cell-type resolution in human islets (11–13). By separating beta cells from other endocrine and non-endocrine cell types, single-cell analysis can test whether T2D-associated changes reflect cell composition, beta-cell state, or both. Many single-cell studies, however, remain mainly descriptive. Differential expression, clustering, and pathway enrichment identify disease-associated genes and programs, but they do not show whether a candidate gene is predicted to perturb a wider beta-cell regulatory network.

Network perturbation analysis addresses part of that gap. scTenifoldKnk reconstructs single-cell gene regulatory networks (14–16) and estimates the transcriptomic consequences of *in silico* knockout of a selected gene (17). In beta cells, this approach can prioritize genes whose perturbation is predicted to affect beta-cell identity, insulin secretory programs, stress pathways, or disease-like transcriptional states. That distinction matters because beta-cell failure may involve both loss of function-maintaining programs and activation of stress-associated or dysfunction-associated programs.

We first reconstructed the beta-cell profile of GSE221156, then built a donor-anchored framework to examine network perturbation. Donor-level pseudobulk analysis supplied a statistically controlled disease signal, and a prior-free genome-wide screen identified expression- and module-based candidates. Independent scTenifoldKnk virtual knockout addressed a different question: which selected genes occupy vulnerable positions in the beta-cell network? Pseudotime, state modeling, and external datasets provided separate lines of context. Together, these analyses allowed us to test when transcript abundance and predicted network criticality converge, and when they part company during T2D beta-cell failure.

## 2 Materials and methods

### 2.1 Dataset collection and analysis overview

Discovery centered on GSE221156. Its official processed beta-cell-only CellxGene object contained 99,029 cells from 48 Demuxlet-resolved donors: 17 ND, 14 PD, and 17 T2D donors (18). In the source workflow, SoupX removed ambient RNA, Demuxlet removed donor multiplets in multiplexed libraries, and Scrublet and DoubletFinder assessed within-library doublets; doublet-enriched mixed-lineage clusters were also removed. Starting from this resource, we modeled beta-cell functional states, performed donor-aware differential expression and prior-free ranking, and then carried out context-matched scTenifoldKnk perturbation, matched-null testing, GRN-scale sensitivity analysis, and external validation.

We used three independent beta-cell datasets for expression validation: GSE153855 (6 ND and 5 T2D donors), GSE81608 (12 ND and 6 T2D donors) (19), and GSE101207 (6 ND and 3 T2D donors). Raw counts from GSE153855 and GSE101207 were summed by donor and analyzed with edgeR quasi-likelihood pseudobulk models. For GSE81608, beta-cell RPKM values were averaged with equal donor weight and tested with limma. Sensitivity models incorporated the available age, sex, BMI, ethnicity, and recovered-cell covariates. Within each cohort, P values were BH-corrected across 12 prespecified genes. Three-cohort evidence was summarized with a two-sided, direction-aware weighted Stouffer analysis; weights were the square root of donor sample size, and the resulting P values were again BH-corrected across the 12 candidates. Cell-level distributions were used only for description. We also examined HbA1c associations in 89 bulk human-islet samples from GSE50244. **Supplementary Table S1** lists which clinical fields were available in each cohort.

### 2.2 Single-cell preprocessing and cell-type annotation

We obtained counts from the official processed GSE221156 resource. Before release, the source study had applied SoupX ambient-RNA correction and multiplet/doublet filtering (18). Cell selection for donor-level inference and network analysis followed the official source-study beta-cell annotations. The working atlas was annotated separately for descriptive visualization after Seurat normalization, variable-feature selection, PCA, graph clustering, and UMAP (20–22). Canonical markers defined the displayed populations, while functional-state labels and module scores summarized beta-cell programs. Neither step altered membership of the official beta-cell GRN input.

### 2.3 Beta-cell subset and disease groups

Two representations of the dataset served different purposes. The 55,791-cell working subset was used for the descriptive atlas and state visualizations. Inferential analyses used the official processed beta-cell-only object, which contained 99,029 cells from 48 donors: 39,484 ND, 35,679 PD, and 23,866 T2D cells. We excluded mixed or incompletely resolved labels from disease-group inference and biological-replicate counts.

### 2.4 Differential expression analysis

For differential-expression inference, we summed raw beta-cell counts from the official Demuxlet-resolved object by biological donor. Genes with nonzero pseudobulk expression were considered expressed and were filtered with edgeR filterByExpr before testing. The primary quasi-likelihood model was ~ sex + scale(age) + scale(BMI) + chemistry + ethnicity + disease. A disease-only model provided a sensitivity analysis. We tested T2D *vs.* ND, PD *vs.* ND, and T2D *vs.* PD contrasts and controlled the false discovery rate by the Benjamini-Hochberg method (23). Statistical significance was defined as FDR <0.05; |log2FC| ≥0.25 was reported separately as an effect-size threshold. Cell-level Wilcoxon results were retained for descriptive continuity, not as biological-replicate inference.

### 2.5 Functional module scoring

Beta-cell functional programs were quantified using curated gene signatures and Seurat AddModuleScore. The modules were beta-cell identity/maturity, insulin biosynthesis and secretion, glucose sensing, mitochondrial oxidative phosphorylation, ER stress/UPR, oxidative stress, dedifferentiation-like state, apoptosis/cell stress, and inflammation. Representative module genes included INS, IAPP, PDX1, NKX6-1, MAFA, UCN3, NEUROD1, NKX2-2, PAX6, RFX6, ISL1, and SLC30A8 for beta-cell identity/maturity; INS, IAPP, GCK, SLC2A2, KCNJ11, ABCC8, PCSK1, PCSK2, SLC30A8, RAB3A, SYT7, VAMP2, and SNAP25 for insulin biosynthesis and secretion; GCK, SLC2A2, KCNJ11, ABCC8, PDX1, MAFA, and SLC30A8 for glucose sensing; OXPHOS genes including NDUFA1, NDUFA2, NDUFB5, NDUFS1, SDHA, UQCRC1, COX5A, COX6A1, ATP5F1A, ATP5F1B, ATP5MC1, and ATP5MC2; HSPA5, XBP1, ATF4, DDIT3, HERPUD1, DNAJB9, ERN1, EIF2AK3, ATF6, and PPP1R15A for ER stress/UPR; TXNIP, HMOX1, SOD2, GPX1, PRDX1, PRDX2, NQO1, GCLC, GCLM, JUN, and FOS for oxidative stress; ALDH1A3, SOX9, CDKN1A, JUN, FOS, MYC, HES1, SOX4, VIM, and LDHA for dedifferentiation-like state; BAX, BBC3, PMAIP1, CASP3, DDIT3, ATF4, GADD45A, and CDKN1A for apoptosis/cell stress; and NFKB1, NFKBIA, STAT1, STAT3, IRF1, CXCL8, CCL2, IL6ST, and TNFAIP3 for inflammation.

Module scores were summarized by disease group and beta-cell state. Spearman correlations between candidate gene expression and module scores were used to link candidate regulators to beta-cell programs.

### 2.6 Candidate regulator prioritization

We reranked genome-wide candidates without a biological-prior term. The primary data-only score used three observed quantities: donor-level pseudobulk differential-expression strength, absolute log2 fold-change magnitude, and the maximum absolute correlation with predefined beta-cell functional modules. Once the prior term was removed, the original data-derived weights (0.35:0.25:0.25) were renormalized to 7/17:5/17:5/17. Regulator and diabetes relevance remained visible as *post hoc* annotations but carried zero weight. We tested equal weighting as well as DE-, effect-size-, and correlation-emphasized alternatives. This prior-free screen was kept distinct from the later virtual-knockout assessment of established beta-cell factors.

data−only score = (7/17)×DE score + (5/17)×|log2FC| score + (5/17)×module−correlation scoreCandidate classes were descriptive. They were assigned from donor-level expression direction together with module association. Downregulated genes linked to identity, secretion, or mitochondrial modules were termed possible function-maintaining candidates; upregulated genes linked to ER stress, oxidative stress, or dedifferentiation modules were termed possible dysfunction-associated candidates. Neither label contributed to the prior-free genome-wide score or to the independent network-evidence score.

### 2.7 scTenifoldKnk virtual knockout analysis

Virtual knockout used scTenifoldKnk version 1.0.3 (17) and the official high-quality GSE221156 beta-cell count matrix. Across all-beta, ND, and T2D contexts, we fixed an outcome-blind universe of 5,000 genes. Donor-balanced pools supplied 1,000 cells to each of 10 independently bootstrapped networks, with separate inference for seeds 101, 202, and 303. Candidate and control knockouts used the same tensor-network and manifold-alignment settings. For each gene, we selected the context with the highest median network-evidence score across the three seeds; the maximum-over-context rule was identical for candidates and controls.

We varied GRN scale in a complete grid. For each of the three seeds, networks contained 200, 500, or 1,000 cells, and each setting comprised 3, 5, or 10 independently inferred networks. Four readouts captured stability: cross-seed whole-profile Z-score Spearman correlation, Top-200 affected-gene Jaccard similarity, concordance across nine prespecified beta-cell programs, and the number of candidate-seed comparisons with finite profile correlations. The primary setting used 1,000 cells and 10 networks. To test high-scale stabilization, we also evaluated 2,000 cells at 10 networks using the same seeds and metrics. The primary analysis retained post-decomposition network weights to two decimal places; otherwise identical three-decimal sensitivity analyses were performed at the 1,000- and 2,000-cell, 10-network settings.

### 2.8 Prior-free network-evidence score

The virtual-knockout evidence score did not include candidate-selection scores or prior knowledge. Within a context, perturbation magnitude combined the maximum absolute Z score (weight 0.45), the mean absolute Z among significant targets (0.35), and the number of FDR-significant targets (0.20). The final score combined rescaled perturbation magnitude (0.45), beta-cell functional-module enrichment (0.30), and overlap with adjusted donor-level T2D versus ND genes, including directional concordance (0.25). We matched candidates by log mean expression, detection fraction, and median log in- and out-strength. Candidate empirical P values counted every unique matched control once and were corrected across 14 candidates using the Benjamini-Hochberg method. For panel-level inference, 200,000 random panels each drew one matched control per candidate, without reusing a gene within a panel. We examined robustness with the panel median, mean candidate-specific percentile, independent matched draws, and leave-one-candidate-out tests.

### 2.9 Beta-cell state and pseudotime analysis

Beta-cell state labels were inferred from module-level programs. A mature program was defined as the average of beta-cell identity/maturity, insulin biosynthesis and secretion, glucose sensing, and mitochondrial OXPHOS module scores. A stress program was defined as the average of ER stress/UPR, oxidative stress, dedifferentiation-like, and apoptosis/cell stress module scores. A dysfunction index was calculated as (stress program - mature program).

Clusters were annotated using mature and dysfunctional ranking scores. The start cluster was selected as the cluster with the highest mature-rank score, and the terminal cluster was selected as the cluster with the highest dysfunctional-rank score. We inferred a deterioration pseudotime axis by projecting each beta cell in the first 10 PCA dimensions onto the vector from the start-cluster centroid to the terminal-cluster centroid and rescaling the projection to the 0–1 range. This axis was used to organize functional-state patterns, not to prove a true temporal lineage (24–26). State-label sensitivity was assessed after removing INS and IAPP from the maturity signatures and excluding cells in the upper 1% of detected-gene count, UMI count, or non-beta-lineage expression score.

### 2.10 External and clinical validation

Candidate regulators were examined in GSE153855, GSE81608, and GSE101207, with the donor as the biological replicate. We considered the T2D *vs.* ND direction, candidate-family FDR, and a direction-aware three-cohort meta-analysis. External expression support required both BH significance in the meta-analysis and agreement of effect direction across all three cohorts. Significant but directionally heterogeneous signals were reported separately. Covariate-adjusted models in GSE81608 and GSE101207 served as sensitivity analyses. Clinical evidence came from GSE50244, which measures bulk human-islet expression alongside HbA1c. Associations in this bulk dataset may arise from beta-cell expression, islet composition, or both.

#### 2.10.1 Validation against independent real-perturbation evidence

We compared selected virtual-knockout candidates with independent perturbation studies, treating the biological sample, mouse, or human donor as the experimental unit. For PDX1, RMA-normalized GSE52258 profiles from FACS-sorted control beta cells, early Pdx1-deficient beta cells, and alpha-cell references (n=5 per group) were analyzed with limma. For MAFA, we analyzed GSE230728 raw counts from 8 WT and 8 beta-cell-specific Mafa-knockout mice using edgeR quasi-likelihood models that included genotype and sex. Mouse genes were mapped to human genes through one-to-one orthologues. scTenifoldKnk Z measures differential network regulation rather than expression fold change, so validation focused on affected-gene enrichment and functional-program affectedness; the Z-sign was not read as an expression direction. Top-50 and Top-200 overlaps were tested with one-sided hypergeometric tests and BH correction across four prespecified candidate-by-set-size comparisons.

For PCSK1, we curated donor-level evidence from CRISPR/Cas9 ribonucleoprotein targeting of PCSK1 regulatory elements in primary human pseudoislets. We found no qualifying public beta-cell or islet genome-wide perturbation transcriptome for PCSK2 or SLC2A2. Their quantitative phenotypes, including rescue and compensation, were therefore extracted from primary pancreatic-islet perturbation studies. Unavailable raw replicates were not reconstructed, and no new P values were calculated. Evidence was classified as genome-wide beta-cell perturbation transcriptomics, direct beta-cell/islet functional perturbation, pancreatic relevance without direct perturbation, or an excluded non-beta-cell context.

### 2.11 Pathway and network visualization

Predicted perturbed genes from virtual-knockout analyses were placed in biological context using GO Biological Process, KEGG, Reactome, and GSEA-style enrichment summaries (27–30). Regulator-centered perturbation networks were visualized for selected core and supplementary candidate genes. Pathway analyses were used to contextualize predicted perturbation effects and were not treated as independent experimental validation.

### 2.12 Statistical analysis

Biological donors, rather than cells, were the replicate unit for differential expression, external validation, and clinical-covariate inference. Discovery edgeR quasi-likelihood tests adjusted for sex, age, BMI, sequencing chemistry, ethnicity, and disease group. GSE153855 and GSE101207 were analyzed by raw-count donor pseudobulk, whereas GSE81608 used equal-weight donor means. Sensitivity models included the available age, sex, BMI, and recovered beta-cell number. External candidate P values were BH-corrected across 12 prespecified genes. We summarized cross-cohort evidence with a two-sided, direction-aware weighted Stouffer test, using square-root donor-sample-size weights and BH correction across the same candidates. Available donor covariates were included in models of candidate expression, modules, state proportions, pseudotime, and dysfunction index; disease-by-sex and disease-by-age terms tested effect modification. The Benjamini-Hochberg method was applied within analysis families, Spearman correlation was used for associations, and FDR<0.05 was the default significance threshold. Single-cell distributions remained descriptive. Real-perturbation transcriptomes used the prespecified limma or edgeR models, while literature-derived quantitative phenotype panels were summarized without new inference.

## 3 Results

### 3.1 The human islet dataset reveals disease-associated beta-cell heterogeneity

We first reconstructed the human islet single-cell profile and annotated major islet cell populations (31–33) (**Supplementary Figure 1**). UMAP visualization separated endocrine and non-endocrine populations, and marker-gene dot plots supported the cell-type annotations. Beta cells formed a clear population in this atlas and were extracted for beta-cell-focused analysis.

Across disease groups, the descriptive beta-cell subset contained 55,791 cells: 18,089 ND, 18,670 PD, 8,240 T2D, and 10,792 with mixed or undetermined labels (**Figures 1A–D**). The functional-module summaries pointed to coordinated remodeling rather than a change in one marker (**Figure 1E**). In donor-adjusted gene models, VIM increased within the dedifferentiation-like module (log2FC +2.479; within-module FDR = 0.00229), CDKN1A increased (+0.673; FDR = 0.0231), SLC2A2 decreased in the glucose-sensing module (-1.429; FDR = 0.00109), and TXNIP increased in the oxidative-stress module (+0.882; FDR = 0.0161). ATP5F1B, UQCRC1, and ATP5F1A had negative OXPHOS estimates, but none passed the within-module FDR <0.05 threshold. We therefore read **Figure 1E** as program-level remodeling with statistical support for specific genes, not as evidence that every member of each module changes significantly.

**Figure 1:**
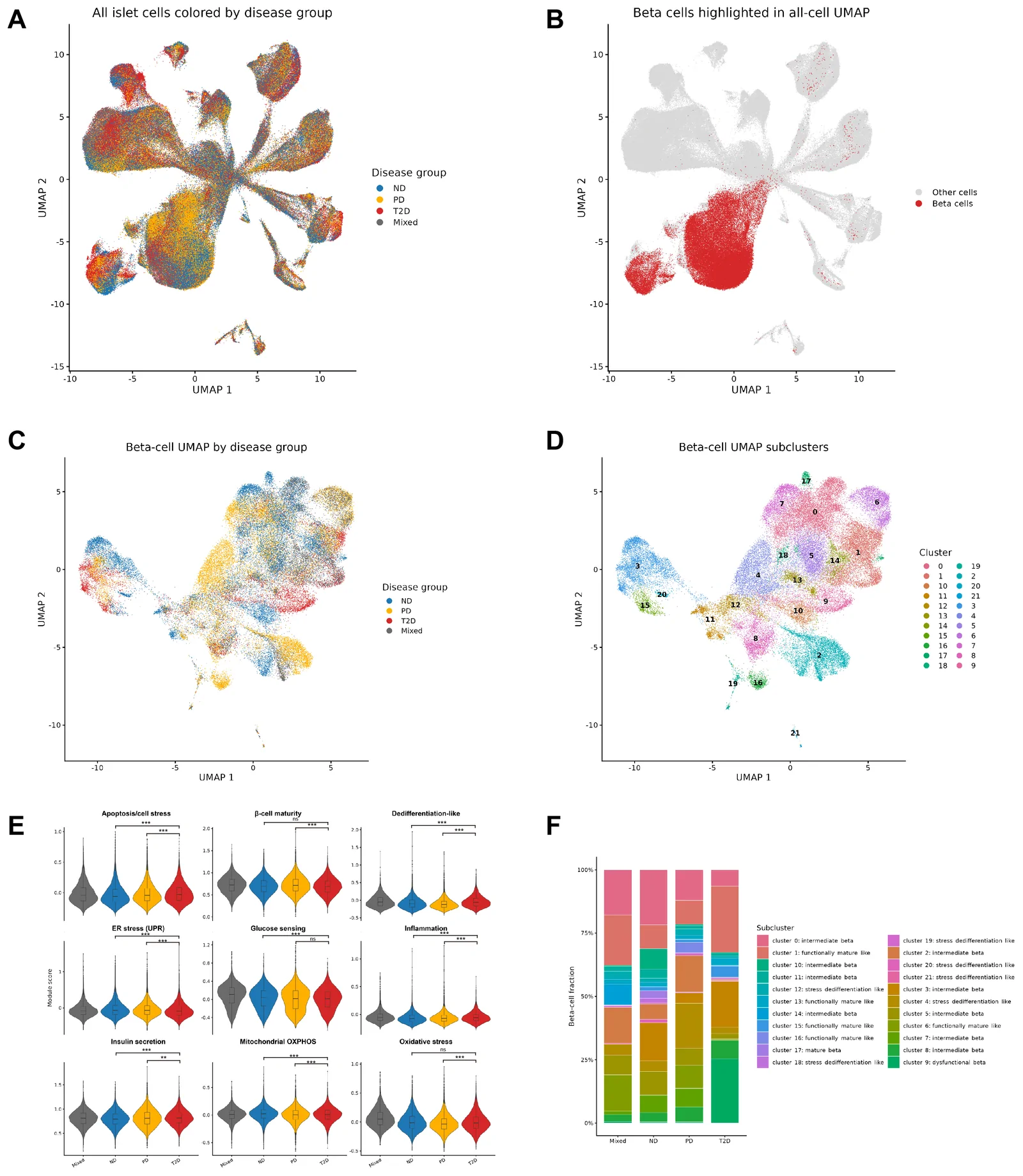
Beta-cell overview and disease-associated functional heterogeneity. **Figure 1** documents descriptive quality characteristics of the processed beta-cell object together with major cell-type annotation of the source islet dataset. **(A)** Violin plots show the distributions of detected genes, UMI counts, and mitochondrial transcript percentages among the 99,029 beta cells retained in the processed GSE221156 object. **(B)** UMAP highlighting beta cells within the full single-cell atlas. **(C)** UMAP of the beta-cell subset colored by disease group. **(D)** UMAP of beta-cell subclusters. **(E)** Disease-level comparison of beta-cell functional module scores. **(F)** Relative beta-cell subcluster composition across disease groups. Compared with the T2D group, **P<0.01, ***P<0.001, and ns P>0.05.

### 3.2 Beta cells show disease-associated transcriptional remodeling and state redistribution

The donor-level pseudobulk analysis resolved a focused disease program. Of 26,164 genes with nonzero pseudobulk expression in the raw beta-cell matrix, 17,989 passed edgeR filtering. The adjusted T2D *vs.* ND contrast identified 674 genes at FDR <0.05, corresponding to 3.75% of those tested; 647 genes (3.60%) also met |log2FC| ≥0.25. Among the significant genes, 356 increased and 318 decreased. Only 9 genes were FDR-significant in PD *vs.* ND, compared with 161 in T2D *vs.* PD. A disease-only sensitivity model identified 1,140 genes for T2D *vs.* ND. This difference was large enough to make the covariate-adjusted model the appropriate primary analysis (**Supplementary Figures S7A–D**; **Supplementary Table S2**).

Module-defined states added resolution to the cell-level picture. Intermediate beta cells remained the largest category in every descriptive disease group. Dysfunctional beta cells, however, represented 2,089 of 8,240 T2D beta cells (25.4%) and less than 1% of cells in the ND, PD, or mixed-labelled groups. These are cell-level percentages, so covariate associations were evaluated with equal donor weight. After BH correction, age and sex were not associated with state proportion, median pseudotime, or median dysfunction index. BMI was positively associated with the dysfunctional-beta proportion at both the ≥50-cell and ≥100-cell donor thresholds (FDR = 0.0231 and 0.0355). The data thus separate a descriptive redistribution of states from the narrower set of donor-level associations. Beta-cell state assignments were unchanged after recalculating the maturity program without INS and IAPP and after conservatively excluding cells in the upper 1% of expression complexity or non-beta-lineage signal; all 14 cluster labels and the mature and dysfunctional endpoint clusters were retained.

### 3.3 Prior-free screening separates disease abundance from network criticality

Removing prior information changed the genome-wide ranking substantially. Across 17,989 tested genes, the original and data-only ranks correlated at Spearman 0.666, yet none of the prior-listed genes appeared in the data-only top 20. ASCL2 ranked 3 and DKK3 ranked 18. By comparison, MAFA, PDX1, and PCSK1 shifted from original ranks 1, 11, and 3 to data-only ranks 176, 332, and 560. The leading data-only candidates were VIM, TBX2-AS1, ASCL2, ELFN1, CELA3A, TBX2, FOXF1, FAIM2, CAMK4, and INHBB (**Figure 2A**; **Supplementary Figures S8A–D**; **Supplementary Table S2**). Results under alternative weighting schemes are shown so that this ranking sensitivity remains visible.

**Figure 2:**
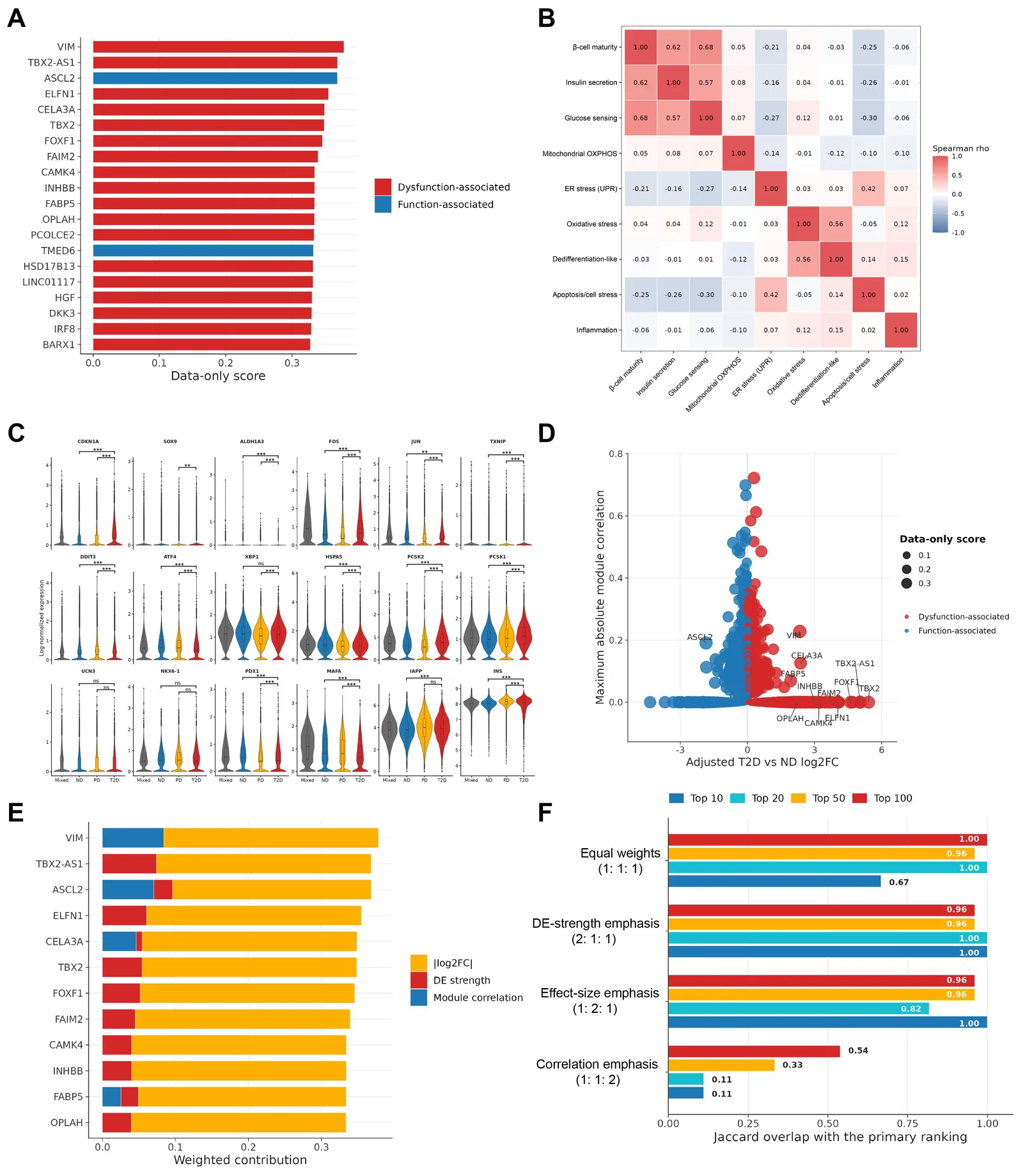
Prioritization of beta-cell candidate regulators. Prior-free genome-wide candidate screening. **(A)** Top 20 genes ranked by donor-level DE strength, absolute effect size, and association with beta-cell functional modules; biological priors have zero weight. **(B)** Correlations among beta-cell functional modules. **(C)** Descriptive single-cell expression distributions for representative beta-cell genes across disease labels; biological-replicate inference comes from the donor-level models. **(D)** Candidate space based on adjusted T2D *vs*. ND log2 fold change, module association, and data-only score. **(E)** Weighted contributions from the three observed-evidence components. **(F)** Jaccard sensitivity of top-N rankings under alternative data-only weights. Established beta-cell factors assessed by virtual knockout form a reference set separate from the prior-free discoveries. Compared with the T2D group, **P<0.01, ***P<0.001, and ns P>0.05.

This reordering does not make the established beta-cell regulators irrelevant; it changes what can be claimed about them. The genome-wide score asks which genes carry abundance- and module-level disease evidence. The independent virtual-knockout analysis asks whether selected beta-cell factors are network-critical. Canonical genes are therefore not presented as unbiased discoveries on the basis of prior relevance. Their support instead comes from virtual-knockout magnitude, functional-module perturbation, cross-seed reproducibility, external datasets, and direct experimental literature. A gene with a modest abundance change may still sit at a vulnerable network position.

### 3.4 scTenifoldKnk virtual knockout prioritizes predicted high-impact perturbation contexts

Expanding the GRNs changed the fine ranking. At 1,000 cells and 10 networks, ATF4, DKK3, SLC30A8, PCSK1, and SLC2A2 ranked first, followed by MAFA, PACSIN2, PCSK2, FFAR4, CDKN1A, NEUROD1, PDX1, ASCL2, and STAT3 (**Figures 3A, B**; **Supplementary Table S3**). Correlation with the initial smaller-scale ranking was negligible (Spearman rho=-0.029), and the selected context changed for 8 of 14 candidates. Network scale and context handling therefore affected ordering within the set more than its overall network signal. The corresponding rank shifts and selected-context changes are shown in **Supplementary Figure S3**.

**Figure 3:**
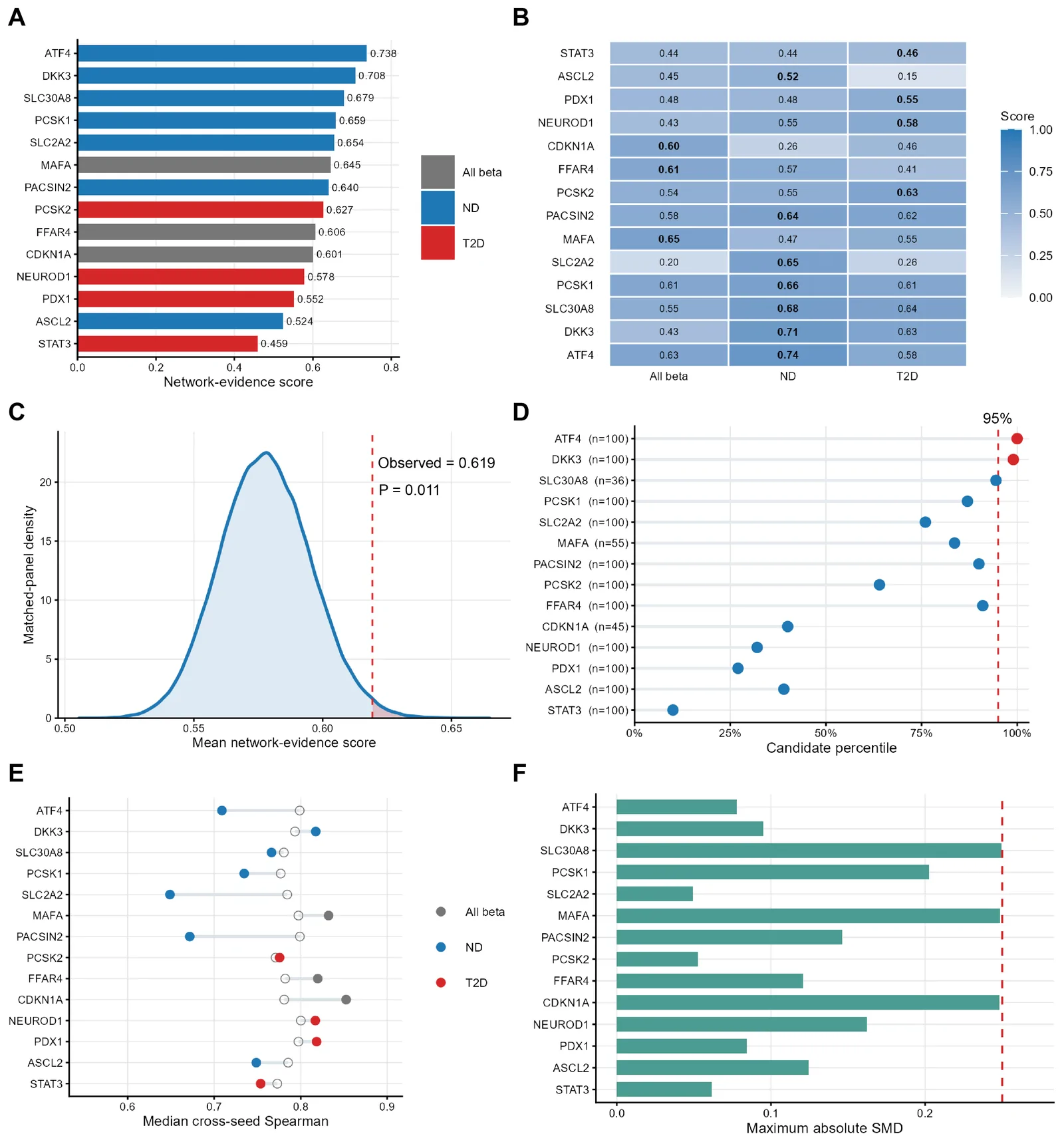
scTenifoldKnk virtual knockout prioritizes functionally relevant beta-cell candidate regulators. Context-selected network evidence and matched-null tests. **(A)** Scores for the fixed 14-gene panel, with color indicating selected context. **(B)** Scores across all contexts; bold values identify the context selected by the candidate/control-common maximum rule. **(C)** Mean-score null from 200,000 matched random panels. The red line indicates the observed mean (0.6193; P = 0.01110). **(D)** Candidate percentiles within finite control pools; none passes Benjamini-Hochberg correction. **(E)** Median cross-seed whole-profile Spearman correlation, with matched-control medians plotted as open circles. **(F)** Maximum absolute standardized mean difference; the dashed line indicates 0.25.

Common support was available for all 14 candidates. Their finite pools contained 36–100 unique controls matched for expression, detection, and network in- and out-strength, and the maximum absolute standardized mean difference did not exceed 0.25 (**Figures 3D, F**). ATF4 and DKK3 gave the smallest finite-pool P values, 0.00990 and 0.01980. After correction across 14 candidates, both had FDR = 0.1386; no individual candidate passed the candidate-family Benjamini-Hochberg threshold.

The panel-level result was different. The fixed 14-gene set had a mean network-evidence score of 0.6193, compared with 0.5779 under the matched-panel null (98.89th percentile; Monte Carlo P = 0.01110; **Figure 3C**). Significance was retained with the panel median (P = 0.00737), the mean candidate-specific percentile (P = 0.01547), and independent candidate-wise matched draws (P = 0.01125). Leave-one-candidate-out P values ranged from 0.00358 to 0.04884. No single gene accounted for the shift.

Larger GRNs were more stable (**Supplementary Figure S6**). Across the 3 × 3 scale grid, median cross-seed whole-profile Spearman correlation increased from 0.265 at 200 cells and 3 networks to 0.884 at 1,000 cells and 10 networks. Median Top-200 Jaccard similarity rose from 0.035 to 0.331, and median functional-program concordance rose from 0.548 to 0.863. The number of candidate-seed comparisons with finite profile correlations increased from 14/42 to 37/42. Extending the 10-network setting from 1,000 to 2,000 cells increased median cross-seed whole-profile Spearman correlation from 0.884 to 0.913 and functional-program concordance from 0.863 to 0.882. Top-200 Jaccard similarity was 0.331 and 0.307, respectively, with overlapping interquartile ranges, supporting stabilization at the 1,000-2,000-cell scale. At 2,000 cells, 34/42 comparisons were finite; the others were structurally zero after sparsification rather than failed runs. Retaining three decimal places yielded finite whole-profile correlations for 39/42 candidate-seed comparisons at both 1,000 and 2,000 cells, compared with 37/42 and 34/42 in the two-decimal primary analysis. Median whole-profile correlations (0.888 and 0.924) and program concordance (0.864 and 0.890) were consistent with the primary stability pattern, showing that the lower two-decimal coverage at 2,000 cells reflected rounding of weak post-decomposition weights rather than failed inference.

The matched analysis identifies a collective network-evidence shift beyond expression abundance and network hubness. Resolution was not uniform. Broad affected profiles and functional programs were more stable than exact downstream targets or small rank differences, while candidate-level statistics distinguished stronger from weaker contributors within the panel.

### 3.5 Core candidate regulators show distinct predicted perturbation-network architectures

We next focused on the four highest-scoring candidates: ATF4, DKK3, SLC30A8, and PCSK1 (**Figure 4**). Their selected-context local networks and affected-gene profiles span distinct biological axes, including the integrated stress response, beta-cell stress/context signaling, zinc transport and insulin crystallization, and proinsulin processing (34).

**Figure 4:**
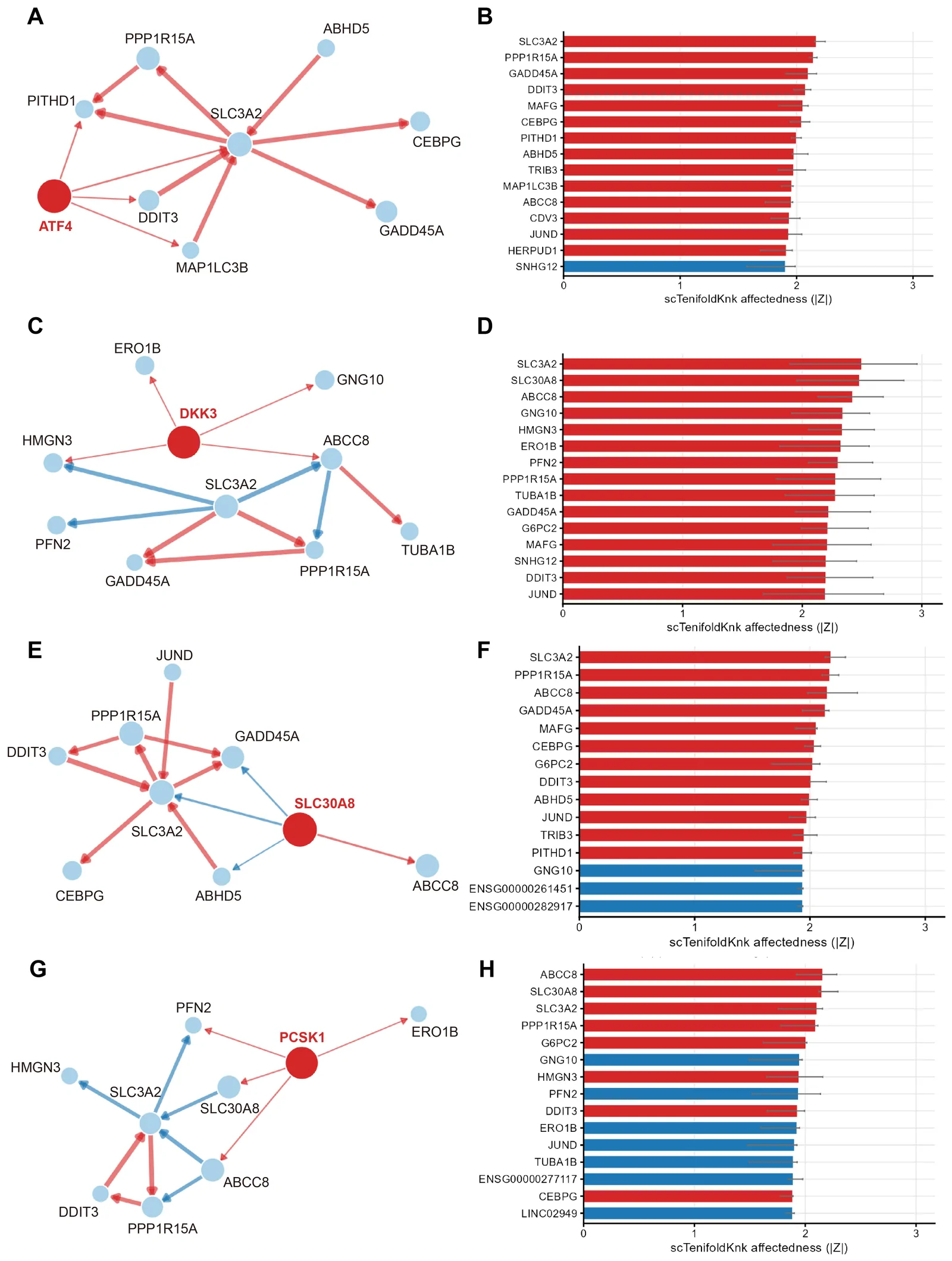
Core candidate regulator-specific perturbation networks. Selected-context networks and affected-gene profiles for ATF4, DKK3, SLC30A8, and PCSK1. **(A, C, E, G)** Sparse networks connecting each candidate with representative highly affected genes. After reciprocal directions were collapsed, twelve representative median wild-type edges across three seeds were retained. Red and blue arrows indicate positive and negative median wild-type weights, respectively, and arrow width is proportional to the absolute median wild-type weight. These edges summarize wild-type structural context. **(B, D, F, H)** Median absolute scTenifoldKnk Z values and seed ranges for the 15 most affected genes. Red marks genes with FDR<0.05 in all three seeds, whereas blue marks the other displayed genes; Z gives the magnitude of predicted network affectedness.

Each of the four candidates produced a distinct three-seed affected-gene profile (**Figures 4A–H**). For ATF4, DKK3, SLC30A8, and PCSK1, the left-hand panels display structural context from median wild-type tensor-network weights. The right-hand panels instead quantify affectedness through median absolute scTenifoldKnk Z, seed ranges, and three-seed FDR status. Thus, network edges describe wild-type structure, whereas Z values report the magnitude of predicted network affectedness. Affected-gene profiles and local network contexts for the other ten candidates are shown in **Supplementary Figures S4**, **S5**.

### 3.6 Pseudotime analysis links candidate regulators to beta-cell state transition

We next placed beta-cell functional modules and candidate genes along an inferred beta-cell deterioration pseudotime. UMAP visualization of pseudotime showed a continuous distribution of beta-cell states, while state annotation separated functionally mature-like, intermediate beta, stress/dedifferentiation-like, and dysfunctional beta cells (**Figures 5A, B**). This analysis linked discrete beta-cell state labels to gradual transcriptional changes, but it should be read as an inferred deterioration axis rather than a direct temporal lineage.

**Figure 5:**
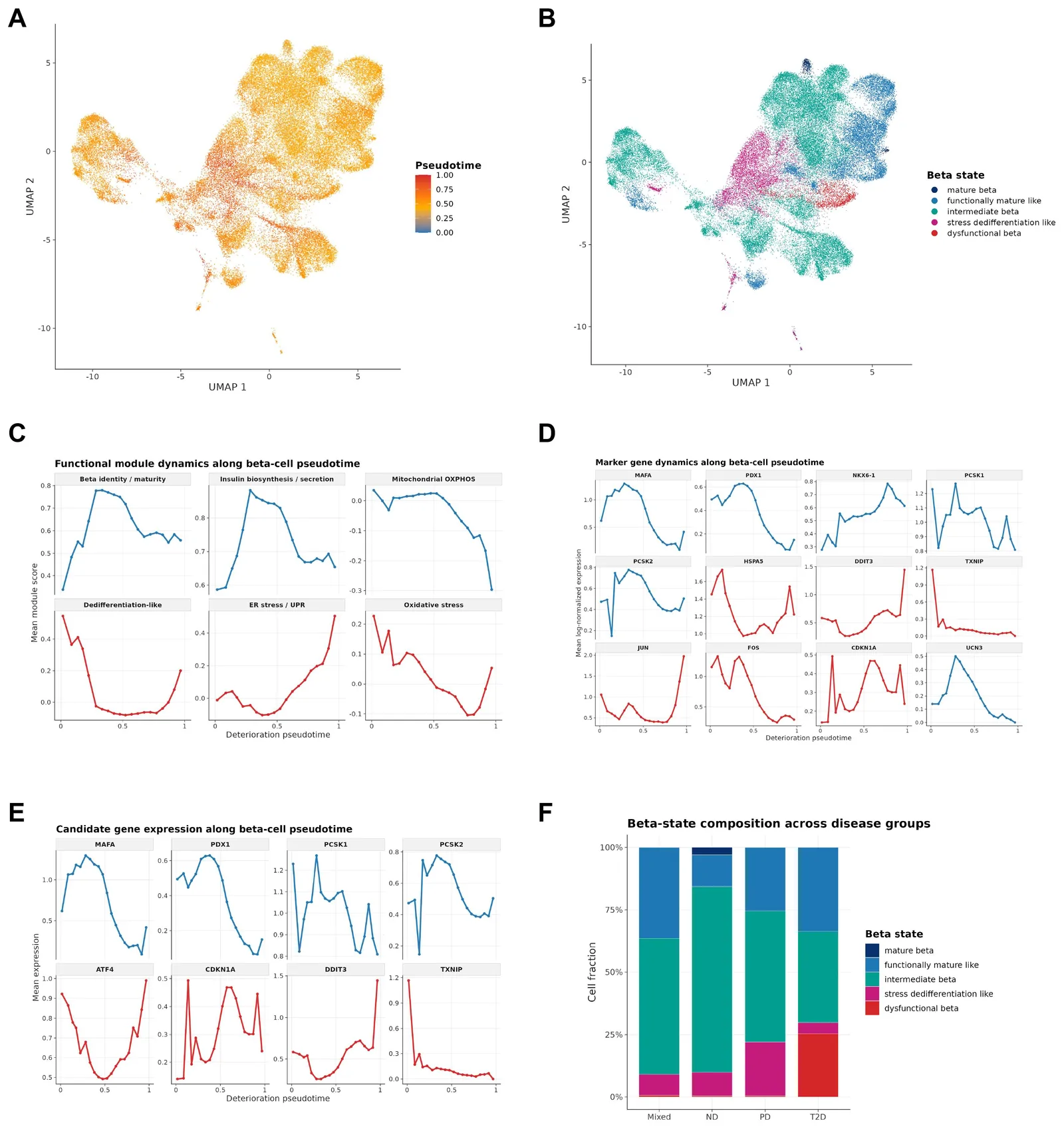
Beta-cell pseudotime and state transition analysis. Image places beta-cell functional states and candidate regulators along an inferred deterioration pseudotime. **(A)** Beta-cell UMAP colored by pseudotime. **(B)** Beta-cell UMAP colored by state annotation. **(C)** Functional module dynamics along pseudotime. **(D)** Marker gene expression dynamics along pseudotime. **(E)** Candidate regulator expression dynamics along pseudotime. **(F)** Relative composition of beta-cell states across disease groups. Compared with the T2D group, *P<0.05, **P<0.01, ***P<0.001, and ns P>0.05.

Functional module dynamics along pseudotime showed coordinated changes in beta-cell programs (**Figure 5C**). Beta identity/maturity, insulin biosynthesis/secretion, and mitochondrial OXPHOS were treated as function-associated programs, whereas dedifferentiation-like, ER stress/UPR, and oxidative stress programs were treated as dysfunction-associated programs. Marker gene dynamics supported the same state-transition pattern. Function-associated genes such as MAFA, PDX1, NKX6-1, PCSK1, PCSK2, and UCN3 showed pseudotime-dependent behavior distinct from stress/dysfunction-associated genes such as HSPA5, DDIT3, TXNIP, JUN, FOS, and CDKN1A (**Figure 5D**). Candidate gene expression dynamics showed opposing functional axes, with MAFA, PDX1, PCSK1, and PCSK2 representing function-associated candidates and ATF4, CDKN1A, DDIT3, and TXNIP representing stress/dysfunction-associated candidates (**Figure 5E**). Disease-group state composition connected these pseudotime patterns to disease status, with T2D showing the strongest enrichment of dysfunctional beta cells (**Figure 5F**).

### 3.7 External and clinical validation support a subset of prioritized candidates

We reanalyzed three external beta-cell cohorts with the donor as the independent unit, avoiding cell-level pseudoreplication (**Figures 6A–E**; **Supplementary Figure S9**; **Supplementary Table S4**). A direction-aware three-cohort meta-analysis identified four BH-significant signals with the same direction in every cohort. DKK3 and PCSK2 increased in T2D (FDR = 9.31×10^–6^ and 4.53×10^-5^), whereas SLC2A2 and ASCL2 decreased (FDR = 0.00186 and 0.00673). CDKN1A was significant overall (FDR = 0.0205), but its direction varied and it was not classified as concordantly replicated. After covariate adjustment, GSE81608 retained all 12 candidate directions (Spearman ρ=0.895), and GSE101207 retained 11 of 12 (ρ=0.930). Adding GSE101207 therefore sharpened the four-gene expression core without concealing between-cohort heterogeneity.

**Figure 6:**
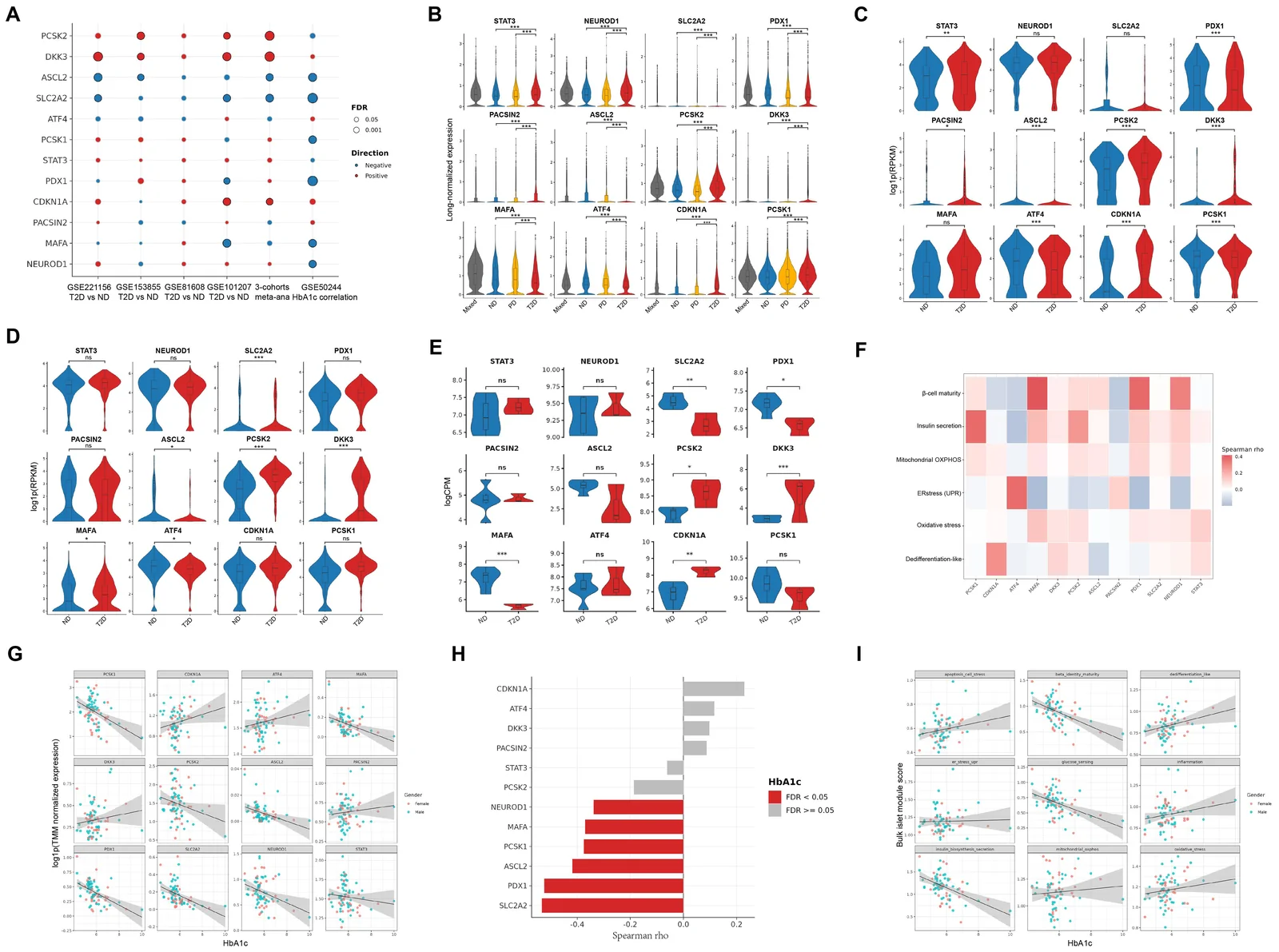
External and clinical validation of candidate regulators. Image assembles candidate-level external and clinical evidence. **(A)** Donor-resolved matrix covering adjusted discovery pseudobulk, GSE153855 and GSE101207 donor pseudobulk, GSE81608 equal-weight donor means, the direction-aware three-cohort meta-analysis, and GSE50244 HbA1c associations. Color indicates direction, point area represents −log10 FDR, and a black outline marks FDR<0.05. **(B)** Descriptive candidate expression across discovery disease labels. **(C, D)** Descriptive cell-level distributions in GSE153855 and GSE81608; cells are not biological replicates. **(E)** GSE101207 donor-pseudobulk logCPM distributions for 6 ND and 3 T2D donors, with each point representing one donor. **(F)** Candidate-module correlations. **(G)** Candidate expression in relation to HbA1c in GSE50244. **(H)** Candidate-level Spearman correlations with HbA1c. **(I)** Associations between functional modules and HbA1c. Formal donor-level statistics appear in panel A, **Supplementary Figure S9**, and **Supplementary Table S4**. Compared with the T2D group, *P<0.05, **P<0.01, ***P<0.001, and ns P>0.05.

We organized expression evidence into four tiers. Tier 1 requires three-cohort meta FDR<0.05 and concordant direction in every cohort. Tier 2 covers corrected meta-analysis support with directional heterogeneity or concordant nominal support; Tier 3 denotes concordant direction without corrected meta significance; Tier 4 is inconclusive. DKK3, PCSK2, SLC2A2, and ASCL2 form the concordant Tier 1 core. CDKN1A is statistically supported overall but remains heterogeneous. Evidence for ATF4, PCSK1, STAT3, PACSIN2, PDX1, MAFA, NEUROD1, FFAR4, and SLC30A8 comes from the combined network, clinical, or perturbation analyses rather than uniform expression replication. Cohort differences are shown explicitly.

Several function-associated genes were also related to glycemic status in GSE50244. PDX1 and SLC2A2 had the strongest negative correlations with HbA1c (Spearman rho approximately -0.525 and -0.534; FDR approximately 5.73x10–^6^ for both); ASCL2, PCSK1, MAFA, and NEUROD1 were negatively associated as well. In discovery-donor models adjusted for disease and technical or clinical covariates, only CDKN1A retained an age main effect (+0.327 logCPM per 1-SD age; FDR = 0.00878), and no candidate retained a sex association. None of 148 disease-by-sex or disease-by-age interaction coefficients for sex survived BH correction. The sole retained interaction was T2D-by-age for mitochondrial OXPHOS (+0.627 module-score units per 1-SD age relative to ND; FDR = 0.0130; **Supplementary Figures S7E, F**; **Supplementary Table S6**). This isolated interaction points to age-dependent disease heterogeneity, not a general age effect.

### 3.8 Orthogonal real-perturbation evidence supports candidate-linked beta-cell programs

Independent perturbation datasets supplied support of uneven strength across candidates (**Supplementary Figures S10**, **S11**; **Supplementary Table S5**). In GSE52258, the response to Pdx1 loss closely followed an alpha-cell reference program (Spearman ρ=0.772 across 15,759 one-to-one orthologues; 83.2% directional concordance), consistent with loss of beta-cell identity. Yet the virtual-KO Top-50 and Top-200 targets were not enriched among Pdx1-KO differential genes (BH q=0.997 for both). Network affectedness is not a gene-by-gene fold-change predictor. In GSE230728, sex-adjusted Mafa knockout showed the expected reduction of Mafa and Slc2a2 together with induction of Gast. Seventy-two Top-200 virtual-KO targets overlapped real-KO differential genes (one-sided P = 0.043), but this signal did not survive correction across four tests (q=0.172). Program affectedness showed moderate descriptive concordance (ρ=0.536 across seven adequately represented programs).

CRISPR targeting of PCSK1 regulatory elements in primary human pseudoislets reduced PCSK1 mRNA and high-glucose insulin secretion while increasing total proinsulin, providing donor-level functional evidence for an insulin-processing phenotype (35). Within the four-gene concordant expression core, global Pcsk2 deficiency impaired prohormone processing. Beta-cell-specific deletion produced a milder glycemic phenotype with reduced mature IAPP, underscoring substrate and model dependence (36–38). Constitutive Slc2a2/Glut2 loss impaired rapid glucose-stimulated insulin secretion and the biosynthetic response to insulin demand. Re-expression of a beta-cell glucose transporter restored secretion, whereas adult beta-cell Glut2 knockdown was milder and accompanied by GLUT1 compensation (39–41). We found no suitable public beta-cell-specific perturbation transcriptome for DKK3 or ASCL2. These two genes remain reproducible candidates for testing, but we do not assign them unsupported causal validation.

### 3.9 Pathway enrichment places virtual-knockout effects in biological context

We mapped robust affected genes from the four highest-scoring candidates to pathways (**Figure 7**). Robustness was defined as FDR<0.05 in at least two of three seeds. Across nine prespecified beta-cell programs, Gene Ontology Biological Process terms, and Reactome pathways, the recurring signals involved beta-cell identity, insulin secretion, glucose sensing, ER stress/UPR, and apoptosis/cell-stress programs. These enrichments locate candidate-specific network affectedness within established beta-cell processes.

**Figure 7:**
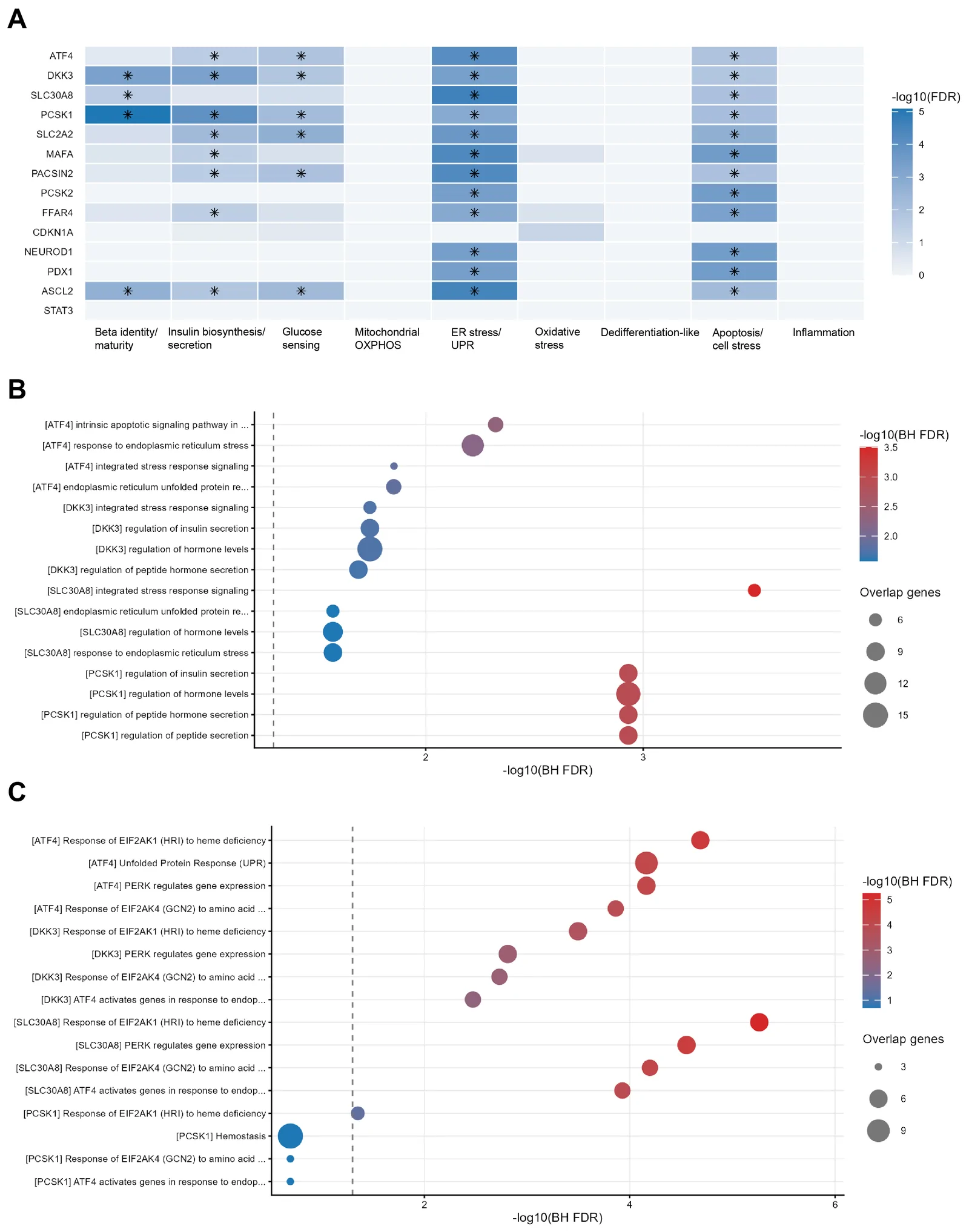
Pathway-level context for scTenifoldKnk perturbation effects. Pathway context for prioritized virtual knockouts. **(A)** Enrichment of nine prespecified beta-cell programs among robust affected genes, defined as FDR<0.05 in at least two of three seeds; stars indicate within-candidate BH FDR<0.05. **(B, C)** Gene Ontology Biological Process and Reactome enrichment for ATF4, DKK3, SLC30A8, and PCSK1. Bubble size indicates the number of overlapping genes, bubble color represents −log10(BH FDR), and dashed lines indicate BH FDR = 0.05. The panels place candidate-specific network affectedness in biological context.

## 4 Discussion

This study joins donor-aware inference with single-cell network perturbation to identify beta-cell vulnerabilities in T2D. Donor-level disease signals anchor the analysis statistically; scTenifoldKnk addresses how gene perturbation may reorganize the beta-cell network. Against controls matched for expression and network strength, the fixed candidate set showed a collective increase in network evidence beyond abundance and connectivity. The framework consequently distinguishes panel-level enrichment from the evidence attached to individual genes.

The state analysis points to an enrichment of a computationally defined dysfunctional beta-cell state in T2D, while PD contains a larger stress/dedifferentiation-like fraction. These cell-level patterns are descriptive. In donor-weighted covariate models, age and sex were not broadly associated with state proportions, pseudotime, or dysfunction index. The independent association between BMI and dysfunctional-state proportion warrants follow-up. Because the states derive from transcriptomic modules and a PCA-based axis, rather than lineage tracing or functional assays, they are annotations of cell state and not evidence of temporal progression.

Discovery and network testing contributed different information. Once the prior term was removed, the genome-wide list changed substantially, confirming the independent role of the prior-free screen. The data-derived network score then combined perturbation magnitude, module enrichment, and adjusted donor-pseudobulk overlap, without carrying forward the candidate-selection score. Its incremental contribution was established by the matched null at the prespecified panel level.

The perturbation studies do not all provide the same kind of support (**Supplementary Figures S10**, **S11**; **Supplementary Table S5**). Pdx1 loss reproduced an alpha-like identity program, but virtual-KO top targets were not enriched among the real-KO differential genes. Network affectedness should therefore not be interpreted as a gene-by-gene expression prediction (42–44). Beta-cell-specific Mafa loss showed target overlap and program concordance that were supportive but correction-sensitive (45, 46). In primary human pseudoislets, PCSK1 CRISPR linked reduced PCSK1 regulation to lower high-glucose insulin secretion and higher proinsulin (35). Published ATF4 and CDKN1A experiments add evidence for stress and survival pathways (47, 48). Taken together, these findings support biological plausibility without experimentally confirming the exact human T2D network edges.

We kept external and clinical evidence stratified rather than assigning each candidate a single validation label. With GSE101207 included, DKK3, PCSK2, SLC2A2, and ASCL2 formed a directionally concordant three-cohort core. Direct islet perturbation links PCSK2 and SLC2A2 to prohormone processing and glucose sensing, while also showing that compensation depends on the experimental model (36–41). DKK3 is notable for convergence among adjusted discovery expression, prior-free ranking, independent network evidence, and meta-analysis despite sparse direct perturbation literature (49). ASCL2 remains a similarly testable beta-cell hypothesis. MAFA and PDX1 make the opposite point: network importance need not coincide with a uniform disease-direction expression change. The Metadichol study used pharmacological induction in PANC-1 cancer/ductal-like cells; overlap in endocrine-identity genes is thematic convergence, not evidence of a shared mechanism or direct validation (50).

GRN scale determined practical reproducibility. Extending the 10-network setting from 1,000 to 2,000 cells increased median cross-seed whole-profile Spearman correlation from 0.884 to 0.913 and program concordance from 0.863 to 0.882, whereas Top-200 Jaccard similarity remained within overlapping interquartile ranges. This pattern supports a high-scale plateau rather than continued material drift. The matched-panel shift remained significant (P = 0.01110) across sensitivity and leave-one-candidate-out analyses. Taken together, these results support a collective candidate-set signal rather than a network-hub artifact. The three-decimal sensitivity analysis restored 39/42 finite comparisons at both high-scale settings without changing the profile- or program-level stability pattern.

Several limitations define the resolution of the study. No individual candidate survived false-discovery correction across the 14 genes, so the panel result does not represent 14 independent gene-level effects. Exact targets and fine rank differences were less stable than broad affected profiles. Direct perturbation is still needed to establish the gene-specific causal effects predicted by scTenifoldKnk.

The most informative follow-up experiments will distinguish network-critical effects from abundance-associated ones. In human beta-cell models, MAFA, PDX1, PCSK1, PCSK2, and SLC2A2 could be perturbed by CRISPR interference or controlled overexpression, with glucose-stimulated insulin secretion, proinsulin processing, calcium flux, and mitochondrial respiration as readouts. CDKN1A, ATF4, DKK3, PACSIN2, and STAT3 could instead be tested under glucolipotoxic, ER-stress, or inflammatory conditions. Single-cell profiling after perturbation would allow the observed downstream programs to be compared directly with virtual-KO predictions. DKK3 is a particularly useful test case because its cross-cohort expression and network evidence currently exceed the available direct experimental support.

## 5 Conclusions

Our donor-anchored framework reveals coordinated remodeling of beta-cell states and regulatory networks in T2D. Prior-free screening captures disease-associated abundance and module signals. Separately, the matched-panel analysis identifies a collective scTenifoldKnk perturbation signal beyond expression and connectivity, with markedly better reproducibility as cell and network counts increase. The result is a statistically supported, experimentally actionable map of candidate regulators and downstream beta-cell programs.

## Data Availability

Publicly available datasets were analyzed in this study. This data can be found here: The discovery and validation datasets analyzed in this study are publicly available from the Gene Expression Omnibus under accession numbers GSE221156, GSE153855, GSE81608, GSE101207, GSE50244, GSE52258, and GSE230728.

## Supplementary material

Supplementary Figure 1 Quality control and major cell-type annotation. **Supplementary Figure 1** documents preprocessing quality and major cell-type annotation for the discovery scRNA-seq dataset. **(A)** Violin plots of quality-control metrics before filtering, including standard single-cell QC features used to remove low-quality cells or potential artifacts. **(B)** UMAP visualization of unsupervised clusters after preprocessing. **(C)** Dot plot of canonical marker genes used to annotate major cell types. **(D)** Major cell-type composition across disease groups, showing whether broad cell-type proportions differ among Mixed, ND, PD, and T2D samples.

Supplementary Figure 2 beta-cell subcluster markers and composition. **Supplementary Figure 2** supports the beta-cell subcluster and state annotations used in **Figure 1** and **Figure 5**. **(A)** Heatmap of beta-cell subcluster marker expression, showing marker genes that distinguish beta-cell subpopulations. **(B)** Relative beta-cell subcluster composition across disease groups, displayed with the recolored disease/state palette.

Supplementary Figure 3 Candidate ranking and selected-context changes after GRN expansion. **Supplementary Figure 3** compares the original 200-cell/3-network screen with the expanded 1,000-cell/10-network analysis. **(A)** Candidate ranks before and after expansion; open circles show the original ranks, filled circles show the expanded ranks, and filled-circle colors indicate the expanded selected context. **(B)** Selected contexts in the original and expanded analyses. Grey, blue, and red denote all-beta, ND, and T2D contexts, respectively; “changed” marks the 8 of 14 candidates whose selected context differed after expansion.

Supplementary Figure 4 Affected-gene profiles for the remaining context-selected candidates. **Supplementary Figure 4** shows the 10 highest-ranked affected genes for the remaining context-selected candidates in the expanded analysis: **(A)** SLC2A2 (ND), **(B)** MAFA (all beta), **(C)** PACSIN2 (ND), **(D)** PCSK2 (T2D), **(E)** FFAR4 (all beta), **(F)** CDKN1A (all beta), **(G)** NEUROD1 (T2D), **(H)** PDX1 (T2D), **(I)** ASCL2 (ND), and **(J)** STAT3 (T2D). Bars show median absolute scTenifoldKnk Z values, and whiskers show the seed range. Red indicates FDR < 0.05 in all three seeds; blue indicates the other displayed genes.

Supplementary Figure 5 Local network context for the remaining context-selected candidates. **Supplementary Figure 5** shows selected-context local networks for the same 10 candidates: **(A)** SLC2A2 (ND), **(B)** MAFA (all beta), **(C)** PACSIN2 (ND), **(D)** PCSK2 (T2D), **(E)** FFAR4 (all beta), **(F)** CDKN1A (all beta), **(G)** NEUROD1 (T2D), **(H)** PDX1 (T2D), **(I)** ASCL2 (ND), and **(J)** STAT3 (T2D). Candidate nodes are red and affected-gene nodes are light blue. Red and blue arrows indicate positive and negative median wild-type weights, respectively; arrow width is proportional to the absolute median wild-type weight. Edges were supported in at least two of three seeds in the expanded 1,000-cell/10-network analysis.

Supplementary Figure 6 GRN profile stability approaches a plateau with increasing cell and network sampling. Median and interquartile range across candidate-seed comparisons are shown for **(A)** cross-seed whole-profile Spearman correlation, **(B)** Top-200 affected-gene Jaccard similarity, and **(C)** concordance across prespecified beta-cell programs. Each measure was evaluated with 200, 500, and 1,000 cells per network and with 3, 5, or 10 independently inferred networks; an additional 2,000-cell point was evaluated at the primary 10-network setting. **(D)** Number of the 42 candidate-seed comparisons with finite profile correlations at each scale setting. Undefined 2,000-cell comparisons reflected structurally zero post-sparsification candidate profiles rather than failed network runs. **(E)** Sensitivity of finite profile coverage to post-decomposition network-weight rounding at the 1,000- and 2,000-cell, 10-network settings. Blue circles show the two-decimal primary analysis; purple squares show the otherwise identical three-decimal sensitivity analysis. Values are proportions of the 42 candidate-seed comparisons with finite full-profile correlations.

Supplementary Figure 7 presents donor-level statistical anchoring and covariate robustness. **(A)** MDS of beta-cell pseudobulk profiles from 48 donors. **(B)** FDR-significant DEG burden for adjusted and disease-only contrasts. **(C)** Volcano plot for the adjusted T2D *vs.* ND pseudobulk analysis. **(D)** Adjusted versus disease-only log2 fold changes. **(E)** Adjusted disease coefficients for leading module-member genes. **(F)** Leading disease-by-sex and disease-by-age interaction estimates with 95% confidence intervals. Only the T2D-by-age interaction for mitochondrial OXPHOS passes within-family BH correction.

Supplementary Figure 8 audits prior removal and the independent network score. **(A)** Original versus prior-free genome-wide ranks. **(B)** Rank changes among canonical beta-cell candidates. **(C)** Empirical cumulative distributions of absolute rank change for genes without a listed prior (n=10,383) and prior-annotated genes (n=34); filled symbols mark the medians. **(D)** Top-N Jaccard sensitivity under alternative data-only weights. **(E)** Components of the independent network-evidence score. **(F)** Relationship between the prior-free expression/module rank and the independent network rank.

Supplementary Figure 9 Donor-aware external validation in three independent beta-cell cohorts. **(A)** Recovered beta-cell counts by donor in GSE153855 (6 ND, 5 T2D), GSE81608 (12 ND, 6 T2D), and GSE101207 (6 ND, 3 T2D); BH-adjusted two-sided Wilcoxon tests are non-significant. **(B)** Donor-level T2D *vs.* ND effects for 12 prespecified candidates. **(C)** Direction-aware weighted Stouffer meta-analysis across three cohorts with BH correction. Red marks FDR-significant signals with full directional concordance; brown marks significant but directionally heterogeneous evidence. **(D)** Covariate sensitivity in GSE81608 and GSE101207. **(E)** Standardized donor-level expression of DKK3, PCSK2, ASCL2, and SLC2A2; symbols reproduce candidate-family FDR. **(F)** Donor-level effects for nine beta-cell functional programs. **(G)** Audit of independent units. GSE101207 is labelled “Donor-level only” because no legacy cell-level inferential result was used. Donors are the biological replicates throughout. Exact P and FDR values are provided in the source data.

Supplementary Figure 10 Independent perturbation evidence for beta-cell programs linked to PDX1, MAFA, and PCSK1. **(A)** Differential expression after early Pdx1 loss in FACS-sorted mouse beta cells (GSE52258; n=5 control and 5 deficient; limma with BH correction). **(B)** Pdx1-KO *vs.* beta and alpha *vs.* beta log2 fold changes across one-to-one orthologues. **(C)** Sex-adjusted differential expression after beta-cell-specific Mafa deletion (GSE230728; n=8 WT and 8 KO; edgeR quasi-likelihood with BH correction). **(D)** Enrichment of virtual-KO Top-50 and Top-200 targets among real-KO differential genes; labels give exact one-sided P and BH q. **(E)** Relative affectedness of nine programs. Pale grey denotes the profile mean, not missingness or absence of an effect. **(F)** Reported donor-level outcome directions after CRISPR targeting of a PCSK1 regulatory element in primary human pseudoislets. NS means that the original study reported no significant difference; it does not establish equivalence.

Supplementary Figure 11 Direct perturbation evidence for PCSK2 and SLC2A2 in pancreatic islets. **(A)** Fold changes in proinsulin-processing endpoints reported for global Pcsk2-null mice or isolated islets; the dashed line is the wild-type reference. **(B)** Proinsulin as a percentage of insulin-like material in Pcsk2-null circulation and pancreas. **(C)** Quantitative endpoints reported for constitutive Slc2a2-null islets and normalized to the corresponding controls. Insulin/DNA was measured in four batches of 60 islets per genotype. Maximal GSIS values are the reported percentages of total insulin content released in five experiments. **(D)** Evidence matrix distinguishing constitutive null, beta-cell-specific or adult knockdown, rescue, and compensatory outcomes. Red denotes an adverse phenotype, grey a preserved or neutral phenotype, blue a rescue, and yellow a compensatory response. Values were taken from published primary reports. Unavailable biological replicates were not reconstructed, and no new inferential P values were calculated. Source data are provided in the accompanying Source Data files.
